# A Pilot Evaluation of the PEACE Implementation Toolkit to Improve the Use of Caregiver Coaching in Early Intervention

**DOI:** 10.3390/bs15091164

**Published:** 2025-08-26

**Authors:** Melanie Pellecchia, Rinad S. Beidas, Liza Tomczuk, David S. Mandell, Aubyn C. Stahmer

**Affiliations:** 1 Center for Mental Health, Psychiatry Department, Perelman School of Medicine, University of Pennsylvania, Philadelphia, PA 19104, USA; david.mandell@pennmedicine.upenn.edu; 2 Department of Medical Social Sciences, Feinberg School of Medicine, Northwestern University, Chicago, IL 60611, USA; rinad.beidas@northwestern.edu; 3 Department of Health Management and Policy, Dornsife School of Public Health, Drexel University, Philadelphia, PA 19104, USA; tomczuk.liza@gmail.com; 4 Department of Psychiatry and Behavioral Sciences, University of California Davis, Davis, CA 95616, USA; astahmer@ucdavis.edu

**Keywords:** early intervention, caregiver-mediated interventions, caregiver coaching, autism spectrum

## Abstract

Caregiver coaching is an essential component of caregiver-mediated interventions for young autistic children. Previous research evaluating usual practice in early intervention (EI) has found that EI providers often do not use caregiver coaching. Increasing the use of caregiver coaching strategies is critical to improving the outcomes of EI. We used a community-partnered process to develop a toolkit of implementation strategies to improve the use of caregiver coaching in EI. This study presents findings from a preliminary evaluation of the toolkit using a non-concurrent multiple-baseline design across groups of providers and caregiver–child dyads. The results indicate that providers’ caregiver coaching fidelity improved following the introduction of the toolkit. Caregivers demonstrated moderate growth in their use of supportive parenting techniques. All providers rated the toolkit as acceptable, appropriate, and feasible. The findings suggest that a toolkit of implementation strategies tailored to support the needs of community-based providers shows promise for improving caregiver coaching in EI.

## 1. Introduction

### 1.1. Caregiver Coaching in Early Intervention

Coaching caregivers to deliver interventions for young autistic children is an evidence-based practice (EBP) for improving child and family outcomes (Landa, 2018). The goal of caregiver coaching is to promote the caregiver’s ability to support their child’s participation in daily activities at home and in their community (Hanft et al., 2004). Coaching is an interactive process between a provider and a caregiver involving observation, reflection, and action. Effective coaching is based on adult learning theory, and it posits that adults benefit from specific strategies that motivate and teach them (Knowles et al., 2020). Caregiver coaching is a multicomponent intervention in which the components are implemented throughout an intervention session with a caregiver and their child. The core components of caregiver coaching are collaboration, practicing the intervention using authentic learning experiences or daily routines, demonstration, practice with feedback, and reflection with problem solving (Dunst & Trivette, 2009; Hanft et al., 2004; Rush & Shelden, 2011).

Infants and toddlers with developmental disabilities, such as autism, are eligible for publicly funded early intervention (EI) services in the United States through Part C of the Individuals with Disabilities Education Act (IDEA). Family-centered care and caregiver coaching are central to the tenets and mandates set forth in Part C of IDEA (Tomasello et al., 2010). This is especially relevant for young autistic children. Intervention initiated at earlier ages for young autistic children, or those with an increased likelihood of autism, improves children’s cognition, language, and development (Boyd et al., 2010; Bradshaw et al., 2014; Fuller & Kaiser, 2020; Noyes-Grosser et al., 2018; Peters-Scheffer et al., 2011; Reichow et al., 2014). Coaching caregivers in intervention delivery bolsters these child gains (Althoff et al., 2019; Bearss et al., 2015; Green et al., 2017). It also improves parental self-efficacy and treatment engagement, and it reduces parental stress (Kasari et al., 2014; Stadnick et al., 2015; Wetherby & Woods, 2006). Caregivers overwhelmingly report that they appreciate being coached (Abouzeid et al., 2020; Siller et al., 2022). Importantly, families of toddlers who receive caregiver coaching use fewer services than families receiving usual community-based services, suggesting significant cost savings (Tsiplova et al., 2022).

### 1.2. Importance of Caregiver Coaching for Autism

Several efficacious caregiver-mediated intervention models have been developed in recent years for young autistic children (Green et al., 2017; Ingersoll & Wainer, 2013; Kasari et al., 2014; Rogers et al., 2019; Wetherby & Woods, 2006). Previous research evaluating the effectiveness of caregiver-mediated interventions for autism have demonstrated gains in social communication and reductions in challenging behavior (Nevill et al., 2018; Ratliff-Black & Therrien, 2021). Research has also shown improvements in parents’ synchrony and children’s communication initiations (Rahman et al., 2016) and joint engagement (Shire et al., 2017) following caregiver-mediated interventions for autism. These models all emphasize coaching as a mechanism to support caregivers in delivering interventions to their children. However, caregiver coaching continues to be underused in community-based early intervention (Aranbarri et al., 2017; Campbell & Coletti, 2013). Observations of community-based EI sessions repeatedly find that EI providers rarely coach caregivers of young autistic children (Douglas et al., 2020; Pellecchia et al., 2022; Salisbury et al., 2012). Instead, EI providers mostly work directly with the child (Campbell & Coletti, 2013; Campbell & Sawyer, 2007). Providers who do coach caregivers tend to implement some, but not all, of the core coaching components during their sessions (Pellecchia et al., 2022).

### 1.3. Research-to-Practice Gap in Caregiver Coaching for Autism

The reasons for this research-to-practice gap are multi-faceted. Previous research evaluating whether EI providers intend to coach parents during their sessions with families of young autistic children found variability in their reported intentions to use caregiver coaching, with some providers reporting strong intentions to coach caregivers and many others reporting that they do not intend to coach caregivers. Further, providers’ intentions to use specific components of coaching (e.g., demonstration and feedback) also varied. Most providers reported that they intended to use some, but not all, of the components of caregiver coaching during their sessions with autistic children (Lawson et al., 2022). Providers’ attitudes toward caregiver coaching (i.e., whether or not they like caregiver coaching), their self-efficacy about coaching (i.e., whether or not they feel confident in their ability to coach caregivers), and their beliefs about group norms related to coaching (i.e., whether or not they believe that other providers are coaching caregivers) are all associated with their intentions to coach caregivers (Lawson et al., 2022). Qualitative studies evaluating providers’ perspectives on caregiver coaching highlight important implementation barriers, including poor self-efficacy, a lack of training, poor group norms among providers related to using coaching, perceptions of parents’ preferences for child-directed intervention, and providers’ beliefs about the fit of coaching with certain families (Albright et al., 2024; Stewart & Applequist, 2019; Tomczuk et al., 2022). Importantly, providers identified social and structural factors, including family characteristics and stigma, that influenced their beliefs about the fit of coaching with families from structurally marginalized groups. Providers often expressed concerns that caregiver coaching is not a good fit for low-income and marginalized or minoritized families (Straiton et al., 2023; Tomczuk et al., 2022). This observed disparity in the use of caregiver coaching for autistic children in community-based settings is likely to exacerbate the disparities observed in the outcomes of young children served in community settings (Hernandez et al., 2025; Pellecchia et al., 2022). These barriers highlight a critical implementation gap, which exacerbates inequities in evidence-based interventions for young autistic children and point to the urgent need to develop targeted implementation strategies to improve EI providers’ use of caregiver coaching within early intervention.

### 1.4. Implementation Science

Implementation science is the study of methods to promote the uptake of research findings and related evidence-based practices into routine clinical care (Eccles & Mittman, 2006). Implementation strategies are approaches or techniques used to improve the adoption, implementation, sustainment, and spread of evidence-based practices (Kirchner et al., 2020). Previous implementation research has highlighted the importance of developing strategies to address specific contextual factors that affect implementation to improve the strategies’ feasibility and effectiveness (Lewis et al., 2018; Powell et al., 2017). Matching implementation strategies to specific contextual needs can change professional behavior and improve evidence-based practice implementation (Baker et al., 2015). Growing evidence suggests that implementation strategies developed in partnership with the constituents who will use them are more effective, accepted, feasible, and likely to be sustained (Concannon et al., 2012; Leeman et al., 2017). There is a critical need to develop and test implementation strategies to increase the use of caregiver coaching for young autistic children who receive publicly funded early intervention services to close the research-to-practice gap in this area.

### 1.5. Purpose of the Current Study

We developed a toolkit of implementation strategies, which we called the Parent Empowerment and Coaching in Early Intervention (PEACE) toolkit, to improve EI providers’ use of caregiver coaching in community-based EI (Pellecchia et al., 2020). The toolkit was developed with frequent input from a community advisory board and was designed to be used in early intervention based on identified implementation barriers to caregiver coaching. The purpose of this pilot study was to evaluate the feasibility, acceptability, and preliminary effectiveness of the PEACE toolkit in improving community-based EI providers’ use of evidence-based caregiver coaching with families of young autistic children. We used a non-concurrent multiple-baseline design across groups of providers and parent–child dyads to achieve this goal. The following research questions were evaluated in this pilot study: (1) Does caregiver coaching fidelity increase following the introduction of the PEACE toolkit? (2) Does caregivers’ use of responsive parenting techniques improve following the introduction of the PEACE toolkit? (3) Do EI providers find the PEACE toolkit feasible and acceptable to use in community-based early intervention? We hypothesized that providers’ fidelity to evidence-based caregiver coaching and caregivers’ use of responsive parenting techniques would improve following the introduction of the PEACE toolkit and that providers would find the toolkit feasible and acceptable.

## 2. Materials and Methods

### 2.1. PEACE Implementation Toolkit

The PEACE toolkit is a theory-informed modular toolkit of implementation strategies that map onto the implementation barriers to caregiver coaching described above (see Table 1). We identified these strategies through an iterative community-partnered process using (1) the Consolidated Framework for Implementation Research (CFIR) (Damschroder et al., 2009) to operationalize the implementation barriers and (2) the Expert Recommendations for Implementing Change (ERIC) taxonomy (Powell et al., 2015) to identify and map relevant implementation strategies onto the identified barriers. A community advisory board (CAB) composed of early intervention providers, administrators, agency leaders, and parents of autistic children participated through regular meetings. CAB members reviewed drafts of the toolkit, provided input on the toolkit as it was developed, and made suggestions for improvement. The CAB provided regular feedback about the toolkit’s fit with early intervention and potential for adoption by early intervention providers.

The PEACE toolkit draws on the theory of planned behavior and targets provider norms (beliefs that others are using coaching), attitudes (whether one likes or dislikes coaching), and self-efficacy (confidence in using coaching) based on the implementation barriers identified through our contextual inquiry and the extant literature (Ajzen, 2020). The toolkit targets group norms by creating social networks and shared experiences through an online communication platform and group facilitation meetings. It targets attitudes through regular messages sent via the online communication platform to increase motivation and modules designed to improve beliefs about the fit of caregiver coaching with families from marginalized backgrounds. PEACE addresses self-efficacy through group facilitation meetings with the auditing of performance, feedback, and reflective discussion.

The PEACE toolkit has three parts: (1) an online resource library; (2) a virtual communication application accessible from any smart device; and (3) weekly group facilitation meetings. The online resource library targets providers’ self-efficacy and attitudes toward coaching. It includes self-paced training modules that cover the foundations of evidence-based caregiver coaching, with specific modules targeting each individual coaching component, and caregiver perspectives on coaching, aligning with caregivers and gaining buy-in for coaching, supporting caregivers from marginalized backgrounds, and improving collaboration and cohesion among providers working with the same child. Each module includes brief videos using examples from community-based EI sessions, easy-to read infographics, tip sheets, and checklists to guide implementation. The library also includes several caregiver-facing modules, with brief, straightforward videos and infographics covering topics such as “What to expect from caregiver coaching,” “What do other parents think about caregiver coaching?” and “Setting expectations for caregiver coaching with your provider.”

A virtual communication application is used to improve providers’ shared norms around the use of caregiver coaching. It facilitates frequent communication, enhances social networks among providers, and creates social norms related to the use of caregiver coaching. Providers can post questions or suggestions, share resources, and receive updates about other providers’ accomplishments. Consultants who are experienced in caregiver coaching frequently post messages and progress updates to increase motivation and shared norms for coaching. The virtual communication includes encouraging messages intended to promote the use of caregiver coaching, positive feedback regarding the use of coaching, prompts to promote engagement, and tips for using coaching. Providers are encouraged to respond to the communication and to post questions. All providers engaged with the communication platform, but we were not able to measure how often they logged on or communicated.

Finally, PEACE includes weekly group facilitation meetings to improve provider self-efficacy and fidelity to caregiver coaching strategies. Facilitation is a multi-faceted implementation strategy that consists of applying a variety of discrete strategies such as audit and feedback and conducting educational meetings through a process of interactive problem solving and support, a shared understanding of a need for improvement, and a supportive interpersonal relationship (Kirchner et al., 2020; Powell et al., 2015). Providers participated in 12 weekly group facilitation meetings with an expert consultant focused on auditing their fidelity to caregiver coaching through video reviews or role-play and performance feedback. The feedback provided during group facilitation was direct positive and constructive feedback regarding the providers’ use of coaching strategies observed in the video reviews and role-play. All providers participated in the video review of their performance during sessions and role-plays during group facilitation. Each group facilitation meeting aligns with the content in the PEACE online resource library modules, is one hour long, and is delivered remotely via videoconference.

### 2.2. Setting

Early intervention sessions were delivered in the family home, with the child and a caregiver present during the entire session. The sessions were approximately one hour long, and families received 1–2 sessions per week throughout the study as part of their usual service delivery. We recorded one of the sessions selected at random each week.

### 2.3. Participants

#### 2.3.1. Early Intervention (EI) Providers

We recruited EI providers from agencies that provide early intervention services to young autistic children and who had received training in Project ImPACT (Ingersoll & Dvortcsak, 2019). Project ImPACT is an evidence-based caregiver-mediated naturalistic developmental behavioral intervention for young children with autism and related disorders. The child-directed interventions are based on applied behavior analysis, focusing on skills related to social communication, play, and behavior, and the intervention includes an explicit focus on caregiver coaching in intervention delivery (Ingersoll & Wainer, 2013; Stadnick et al., 2015). We selected providers who were trained in Project ImPACT to ensure a common baseline level of knowledge about caregiver coaching and autism intervention. Agency directors sent information about the study to their staff, and interested staff contacted the study team who then initiated the informed consent process. Interested providers were enrolled if they had (1) completed training in Project ImPACT and (2) provided early intervention services to families of young autistic children.

#### 2.3.2. Parents and Children

Families were recruited from the participating EI providers’ caseloads. Providers shared information about the study with families on their caseload. Interested families contacted the study team and were screened for eligibility, and study participation was explained in detail. One family was enrolled into the study from each providers’ caseload. Families completed the informed consent process upon enrollment. The inclusion criteria for families were as follows: (1) a child with an autism diagnosis or identified as having an increased likelihood of autism by the EI system (i.e., screened positive on the MCHAT and clinical observation by the EI system indicated autism likelihood), (2) who was 33 months of age or younger upon enrollment, and (3) was receiving early intervention services from an enrolled provider and (4) a caregiver who spoke English or Spanish.

### 2.4. Measures

#### 2.4.1. Caregiver Coaching Fidelity

Caregiver coaching fidelity was assessed using the PEACE Caregiver Coaching Fidelity Tool (Pellecchia et al., 2022) on video-recorded observations of EI sessions. The tool comprises 25 items rated on a 5-point scale, with a rating of 4 or 5 indicating acceptable fidelity. The PEACE Caregiver Coaching Fidelity Tool was developed to assess the use of the core components of evidence-based caregiver coaching (collaboration, practicing in daily routines, demonstration, practice with feedback, and reflection with problem solving). It provides an overall fidelity score, as well as scores for fidelity to each component. The item definitions and coding scheme were based on other measures of family-centered early intervention and adapted to include the core components of adult learning theory-based caregiver coaching (Basu et al., 2010). It has been used to evaluate caregiver coaching fidelity for EI providers working in community-based EI systems (Pellecchia et al., 2022). Consistent with conventional fidelity metrics (Borrelli et al., 2005), a score of 80% indicates acceptable adherence. Providers’ use of caregiver coaching was assessed using video recordings of EI sessions with their enrolled parent–child dyad. One EI session was recorded in its entirety each week throughout the study period. Trained research assistants masked to group assignment coded the videos. The coders were trained through didactic instruction with practice and feedback until they achieved at least 90% accuracy on three consecutive training videos. Inter-observer agreement was coded for 20% of the videos, evenly distributed across providers and study phases.

#### 2.4.2. Intervention Acceptability, Appropriateness, and Feasibility

The acceptability, appropriateness, and feasibility of the PEACE toolkit were measured using the Acceptability of Intervention Measure (AIM), Intervention Appropriateness Measure (IAM), and Feasibility of Intervention Measure (FIM) (Weiner et al., 2017). The AIM, IAM, and FIM are four-item measures of implementation outcomes that are leading indicators of implementation success. They have strong content validity, discriminant content validity, reliability, structural validity, structural invariance, known-groups validity, and responsiveness to change. Cronbach’s alphas for the scales reported in previous studies were 0.85 for the AIM, 0.91 for the IAM, and 0.89 for the FIM (Weiner et al., 2017). EI providers rated their views on the acceptability, appropriateness, and feasibility of the PEACE toolkit using the AIM, IAM, and FIM after completing the twelve weeks of group facilitation.

#### 2.4.3. Caregiver Responsiveness

Caregiver responsiveness was assessed using the Parenting Interactions with Children: Checklist of Observations Linked to Outcomes (PICCOLO: Roggman et al., 2013). We evaluated changes in caregiver responsiveness as a measure of improvements in caregivers’ use of the intervention techniques learned during the coaching sessions. The PICCOLO is a checklist of 29 observable, developmentally supportive parenting behaviors with children ages 10–47 months; it has been used to measure changes in caregiver responsiveness with caregivers of children with autism and developmental disabilities until five years of age (Alquraini et al., 2019; Vilaseca et al., 2019). It is a strengths-based measure of parenting interactions that predicts children’s early social, cognitive, and language development with solid psychometrics. It has been successfully used to assess changes in parent responsiveness with families of children with disabilities and from diverse ethnic backgrounds (Roggman et al., 2013; Vilaseca et al., 2019), and it was found to be sensitive to changes with large effect sizes in a community-based sample of families receiving Project ImPACT (Stahmer et al., 2020). The PICCOLO has four domains: Affection, Responsiveness, Encouragement, and Teaching. The total scores on each domain are used to monitor changes in parenting behaviors. A brief (10 min) parent–child interaction was video-recorded at baseline and after 12 weeks of intervention to assess changes in parent responsiveness. Members of the research team masked to groups and time points scored the checklist from the video-recorded interaction.

### 2.5. Design and Analyses

Changes in providers’ caregiver coaching fidelity were assessed using a single-case non-concurrent multiple-baseline design across three groups of EI providers and parent–child dyads (three providers per group). A non-concurrent multiple-baseline design was selected to improve the feasibility of evaluating the intervention across groups of providers in a community-based service setting. While the staggering of interventions contemporaneously across participants in a multiple-baseline design is usually recommended to improve rigor and causal inferences in single-case research designs, the use of non-concurrent multiple-baseline designs has been recommended for use in community-based service settings, schools, and designs comparing groups of participants where a concurrent multiple-baseline design is not feasible (Harvey et al., 2004; Slocum et al., 2022). Consistent with best practices in non-concurrent multiple-baseline designs and to improve rigor, providers were randomly assigned to groups, and the groups were randomized to the length of the baseline phase (Harvey et al., 2004; Watson & Workman, 1981). All intervention sessions across phases were conducted once per week with each family. The baseline phase lengths were selected to be consistent with best practices in single-case research design, with a minimum of three baseline observations. The baseline phases were as follows: Group 1 was randomized to 3 baseline sessions, Group 2 was randomized to 5 baseline sessions, and Group 3 was randomized to 4 baseline sessions. Following baseline, providers received access to the PEACE online modules and online chat, and they participated in twelve weekly group facilitation sessions. Each weekly group facilitation session was one hour long and was held via teleconference. Providers implemented their sessions as usual with their enrolled family dyads during the twelve weeks of intervention. We video-recorded one session each week with each provider–family dyad during the twelve weeks of intervention. There was no provider overlap across groups during group sessions to ensure independence among groups. The sessions were video-recorded using teleconferencing software.

Following best practices in visual analysis for single-case research design, we evaluated the level, trend, variability, immediacy of effect, overlap, and consistency of data patterns across phases for all groups and used the systematic protocol for visual analysis of multiple-baseline design graphs developed by Wolfe et al. (2019) to determine whether a functional relation was present in the data. We evaluated the overlap in data across phases for each group using the percentage of non-overlapping data (PND) for single-case research design (Carr, 2015; Schlosser et al., 2008; Scruggs & Mastropieri, 1998). We calculated effect size estimates for changes in coaching fidelity across phases for each group using the log response ratio. The log response ratio is an effect size index commonly used in single-case research design to quantify the change from phase A to phase B in proportionate terms (Pustejovsky, 2015; Pustejovsky, 2018). In addition to examining changes in overall fidelity, we examined changes in fidelity to each coaching component (collaboration, daily routines, demonstration, feedback, and reflection and problem solving) using the percentage of non-overlapping data across phases for each group. Caregiver responsiveness was evaluated at baseline and after 12 weeks of intervention. Providers’ perspectives on the acceptability, appropriateness, and feasibility of the PEACE toolkit were measured following 12 weeks of access to the implementation strategies. Descriptive and summary statistics for caregiver responsiveness and acceptability, appropriateness, and feasibility scores were calculated.

## 3. Results

### 3.1. Participant Demographics

#### 3.1.1. Providers

Eight EI providers participated. There were three providers in both Groups 1 and 3 and two providers in Group 2. All providers were female, five were White, and three were Black. All providers had graduate degrees (Table 2). Provider demographics were similar to characteristics reported in previous samples with this population (Lawson et al., 2022; Pellecchia et al., 2022).

#### 3.1.2. Caregivers

Eight caregivers, one caregiver from each enrolled provider’s caseload, participated. All of the caregivers were female, three (37.5%) were White, three (37.5%) were Hispanic, two (25.5%) were Black, one (12.5%) was Asian, and two (25%) reported “Other” as their race or ethnicity. The caregivers’ highest levels of education were high school diploma or GED (2), some college (2), or a college degree (4). The annual household incomes were as follows: four (50%) of the families had an annual household income of less than USD 20,000, one (12.5%) caregiver reported an annual household income of USD 20–40,00, one (12.5) caregiver reported an annual household income of USD 40–60,000, and two of the families (25%) had an annual household income of over USD 60,000. All caregivers received services in English. See Table 2.

### 3.2. Inter-Observer Agreement

The mean inter-observer agreement balanced across providers and sessions for PEACE fidelity coding was 83% (range = 61–95%). The mean inter-observer agreement for PICCOLO observations was 81% (range = 79–86%). Consensus and further review by a master coder were used for any inter-observer agreement scores below 80%.

### 3.3. Overall Caregiver Coaching Fidelity

**Figure 1:**
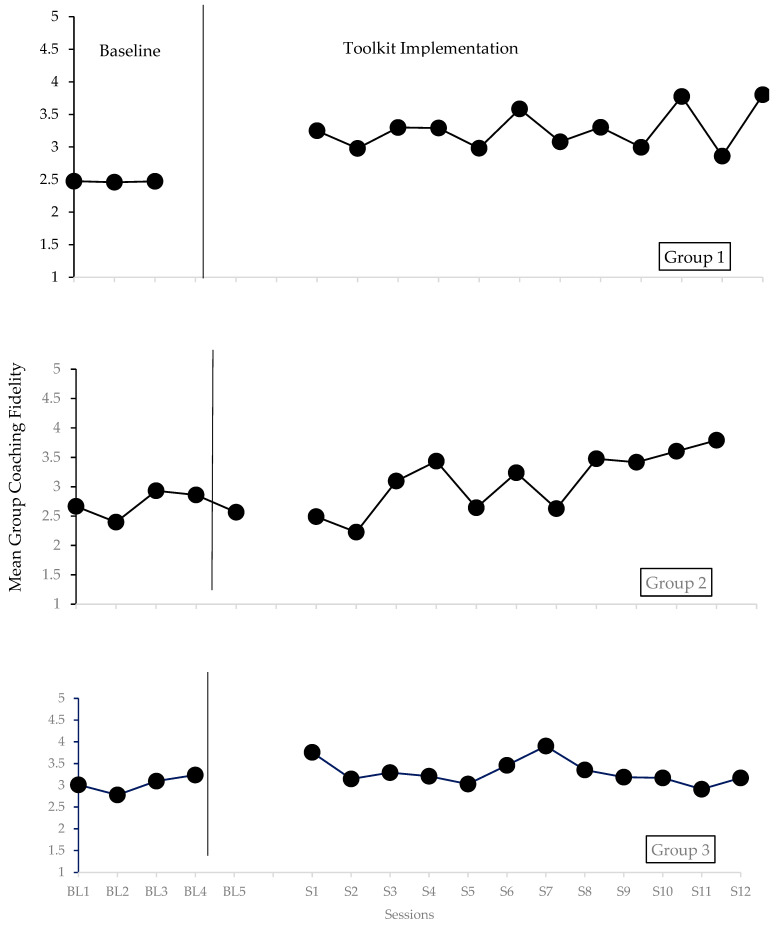
Mean coaching fidelity for groups of providers at baseline and after introducing the PEACE toolkit.

The mean coaching fidelity for providers in each group at baseline and following the introduction of the PEACE toolkit is depicted in Figure 1. Following best practices in visual analysis for single-case research design, we evaluated the level, trend, variability, immediacy of effect, overlap, and consistency of data patterns across phases for all groups (Kratochwill et al., 2010).

#### 3.3.1. Level

Visual analysis of the data indicated stable levels of data during baseline and intervention phases for all three groups.

#### 3.3.2. Trend

Increasing trends in the data were observed during the intervention phase for Groups 1 and 2. However, the data trend was flat during the intervention phase for Group 3.

#### 3.3.3. Variability

Visual inspection of the data indicated stability in the data for all three groups, with little fluctuation around the trend line.

#### 3.3.4. Immediacy of Effect

An immediate change from the last three data points at baseline to the first three datapoints in the intervention phase was observed for all three groups, demonstrating an increase in caregiver coaching fidelity.

#### 3.3.5. Overlap

The percentage of non-overlapping data (PND) for single-case research design was used to evaluate the overlap in data across phases for each group. The PND for Group 1 was 100%, indicating that the PEACE toolkit improved Group 1 providers’ overall caregiver coaching fidelity (Highly Effective). The PND was 73% for Group 2 (Effective) and 64% for Group 3 (Questionable).

#### 3.3.6. Consistency of Data Patterns Across Phases

The data patterns of the treatment phases were similar for Groups 1 and 2, with immediate changes in the data observed following the introduction of the toolkit and subsequent increasing trends. However, the data for Group 3 were inconsistent with those of the other groups and demonstrated a flat trend during intervention.

#### 3.3.7. Functional Relation

The total score generated by the systematic protocol for visual analysis of multiple-baseline design graphs (Wolfe et al., 2019) was 3.54, indicating that a functional relation was present in the data.

#### 3.3.8. Effect Size

We used the log response ratio to estimate effect sizes for changes in caregiver coaching fidelity across phases for each group. The results indicated a very small to small effect for each group: Group 1 = 0.28, Group 2 = 0.14, and Group 3 = 0.09.

### 3.4. Changes in Caregiver Coaching Fidelity for Coaching Components

Changes in fidelity to each coaching component for the groups of providers are presented in Table 3. Significant improvements in providers’ fidelity to collaborative coaching strategies were observed in all three groups (Group 1: PND = 100%, *p* = 0.001; Group 2: PND = 82%, *p* = 0.003; Group 3: PND = 78%, *p* = 0.01). Significant improvements in EI providers’ fidelity to coaching within families’ daily routines were observed for Group 1 (PND = 100%, *p* = 0.001). No significant changes in coaching in daily routines were observed for providers in Group 2 (PND = 36%, *p* = 0.15) or Group 3 (PND = 44%, *p* = 0.14). No significant changes in providers’ fidelity to demonstration coaching strategies were observed in any of the three groups (Group 1: PND = 50%, *p* = 0.16; Group 2: PND = 0%, *p* = 1.0; Group 3: PND = 11%, *p* = 0.57). Significant improvements in providers’ use of feedback coaching strategies were observed for Group 1 (PND = 83%, *p* = 0.01) and Group 2 (PND = 73%, *p* = 0.01), and moderate but not significant improvements in fidelity to feedback coaching strategies were observed for providers in Group 3 (PND = 56%, *p* = 0.07). Significant improvements in providers’ fidelity to reflection and problem solving coaching strategies were observed across all three groups (Group 1: PND = 100%, *p* = 0.001; Group 2: PND = 73%, *p* = 0.01; Group 3: PND = 78%, *p* = 0.01). The overall coaching fidelity ratings for each provider during the first baseline observation session and the final intervention session are displayed in Table 3.

### 3.5. Acceptability, Appropriateness, and Feasibility

All providers rated the PEACE toolkit as highly acceptable, appropriate, and feasible for use within early intervention, with a mean rating of 5 out of 5 for all three measures across all providers.

### 3.6. Changes in Caregiver Responsiveness

Caregivers demonstrated slight improvements in their use of supportive parenting techniques during the second observation for most domains. No change was observed in caregivers’ use of parenting techniques in the Affection domain; however slight improvements were observed in caregivers’ use of parenting techniques in the Responsiveness, Encouragement, and Teaching domains of the PICCOLO. The mean scores for each PICCOLO domain for caregivers at baseline and after 12 weeks of intervention are displayed in Table 4.

## 4. Discussion

This pilot study evaluated the feasibility, acceptability, and preliminary effectiveness of the PEACE implementation toolkit. The PEACE implementation toolkit was designed to improve the implementation of caregiver coaching in publicly funded early interventions for families of young autistic children. We used a community-partnered, iterative development process to develop an implementation toolkit that mapped onto identified implementation barriers to caregiver coaching. The results of this pilot study indicate that the toolkit was highly acceptable to community providers and is feasible to implement in community-based early intervention. Findings from the non-concurrent multiple-baseline design across groups of providers and caregiver–child dyads indicate that the PEACE toolkit shows promise for improving providers’ use of evidence-based caregiver coaching.

Interestingly, all providers reported having received training in parent coaching before the study, yet most providers demonstrated subthreshold coaching fidelity at baseline. This subthreshold level of intervention fidelity, coupled with the variability in fidelity observed across providers at baseline, mirrors the variability in coaching fidelity present following traditional training models. This is also consistent with previous research that found that training alone is not sufficient to change practice for most providers (Beidas et al., 2012). Implementation studies have repeatedly demonstrated that evidence-based practices are under-used, even when practitioners have received considerable training (Bond et al., 2014), further highlighting the need for targeted implementation support.

All providers who participated in this pilot study rated the PEACE toolkit as highly feasible, acceptable, and appropriate for use in early intervention. We developed the PEACE toolkit in partnership with community constituents, and we tailored the strategies to meet the unique contextual needs of community-based early intervention. These high ratings of feasibility, acceptability, and appropriateness highlight the effectiveness of the community-partnered development process and are consistent with previous implementation research showing the importance of developing implementation strategies in partnership with community constituents to ensure that they are feasible and appropriate for the setting and address specific barriers where the intervention is being delivered (Jones et al., 2018). Although beyond the scope of this project, it would have been helpful to conduct follow-up qualitative interviews with providers to gain in-depth information about their perspectives on the PEACE toolkit overall, as well as feedback for areas of potential improvement. Future research that includes a qualitative inquiry into providers’ experiences with using the toolkit can inform any potential adaptations for large-scale implementation.

Following the introduction of the PEACE implementation toolkit, visual analysis indicated improvements in overall coaching fidelity for two out of the three groups. However, the effect size estimates for overall caregiver coaching were small. Some individual providers did not demonstrate sizeable improvements in their overall coaching fidelity. Consistent with usual large-scale training efforts, the PEACE toolkit was administered in a group format, with all providers receiving the same amount of implementation support. Our pilot data indicated that a tiered approach to delivering implementation support based on provider needs, rather than providing a uniform level of implementation support to all providers, may be necessary for improving providers’ caregiver coaching. For example, providers who work in an agency that provides regular support for caregiver coaching, have many years of experience coaching caregivers, or buy-in to a caregiver-mediated intervention approach may not require intensive implementation support. However, some providers will need individual and targeted support to implement caregiver coaching with fidelity. Tailoring implementation strategies based on providers’ individual characteristics and needs has been identified as an effective and efficient approach to improving the implementation of evidence-based practices (Powell et al., 2017). This pilot study’s mixed findings, with some providers requiring additional implementation support to reach fidelity, should be used to inform future adaptations to the PEACE toolkit. Future research should include a systematic evaluation of the effectiveness of individualized implementation support based on each provider’s identified implementation barriers. This could include more intensive performance feedback, motivational interviewing, and collaborative problem solving with the provider to overcome implementation barriers.

In addition to examining the change in overall fidelity, we examined the change in fidelity to each coaching component (collaboration, daily routines, demonstration, feedback, and reflection and problem solving). Providers varied in their overall coaching fidelity. Among those with poorer fidelity, lows scores were driven by different coaching components. This finding is consistent with previous observations, in which providers’ coaching fidelity varied across components (Pellecchia et al., 2022). Notably, individual providers varied in their fidelity to coaching in daily routines. Supporting caregivers in their daily routines is consistent with recommendations for family-centered care in early intervention (DEC, 2014; Woods et al., 2004). Yet EI providers consistently struggle to implement interventions with caregivers within their naturally occurring routines (Campbell & Sawyer, 2007; Pellecchia et al., 2022; Sawyer & Campbell, 2009). Most evidence-based interventions for young children with autism and related disorders emphasize play-based interventions that use the child’s interests to guide the interaction. It is possible that these approaches conflict with supporting caregivers in daily routines (e.g., getting dressed may not be a preferred activity for many young children), which may inhibit EI providers’ ability to coach in daily routines. Additionally, previous qualitative research exploring EI providers’ perspectives on coaching also indicated that they feel uncomfortable coaching in daily routines as opposed to play-based interactions, which likely leads to their minimal use of coaching in daily routines (Tomczuk et al., 2022). Similar challenges with fidelity to demonstration coaching techniques were observed across groups of providers. The low fidelity to demonstration coaching techniques observed for most providers is consistent with previous evaluations of early intervention providers’ coaching (Artis et al., 2022), and it highlights the difficulty of implementing this coaching technique. It is also possible that providers’ use of and training in child-directed intervention strategies, rather than coaching caregivers to use intervention strategies, can inhibit their use of demonstration. This is consistent with previous observations of early intervention in which EI providers were more likely to intervene directly with the child rather than coach caregivers (Campbell & Coletti, 2013). Despite the challenges with increasing fidelity to coaching in daily routines and demonstration for most providers, positive changes in other coaching components were observed after exposure to the PEACE implementation toolkit. Improvements in the use of feedback, collaboration with caregivers, and reflective problem solving were observed for most providers. These improvements observed for most, but not all, coaching components indicate that additional targeted support may be needed for more complex coaching components with some providers. It is important to highlight the variability in the change in caregiver coaching observed both across providers and across individual coaching components. These findings suggest that some providers may need more targeted or individualized implementation support to effect change in their use of caregiver coaching. Previous research has indicated the need for tiered levels of implementation support based on individual practitioner needs, with some, but not all, providers requiring more intensive consultation in order to meet the required fidelity benchmarks (Brock et al., 2021). Future research that evaluates the effectiveness of tiered levels of implementation support, delivering more intensive training and consultation for providers who fail to meet the required benchmarks, can offer an innovative approach to increasing the wide-scale use of caregiver coaching among EI providers.

We evaluated the change in caregivers’ use of responsive interaction techniques using the PICCOLO, a standardized direct observation measure. Slight improvements in the use of responsive interaction techniques were observed across the groups of caregivers after twelve weeks of intervention, but, overall, the changes were not significant. It is possible that the constructs measured in the PICCOLO did not align well with the intervention strategies taught during the early intervention sessions. It is also possible that a period of intervention longer than twelve weeks may be needed to effect significant change in caregivers’ use of the targeted intervention techniques. Future research should evaluate the sensitivity of the measures used to evaluate the changes in caregivers’ responsiveness, as well as the length of intervention time needed to effect change in these caregiver behaviors.

This project was implemented in partnership with community service providers who serve children in community settings. This community-based research presents a series of unique facilitators and barriers. The providers who participated in this pilot study were motivated and engaged throughout the process. A strong community–academic partnership was integral to the success of this project and likely served as an integral implementation facilitator. Notable barriers to implementation included scheduling conflicts and challenges with coordinating group facilitation sessions across providers.

Several limitations are worth noting. First, this study included a small sample of EI providers and caregivers. Although the sample is consistent with that adopted in single-case designs and pilot studies, its small nature limits the generalization of the findings to other groups of providers and families. It is also important to note that we observed variability in baseline coaching fidelity across groups, with Group 3 demonstrating higher baseline coaching fidelity and, subsequently, smaller increases in fidelity after the PEACE toolkit was introduced. It is difficult to evaluate the effects of the toolkit with providers who already demonstrate higher levels of baseline fidelity. Lastly, our ratings of acceptability, feasibility, and appropriateness were very high, demonstrating possible ceiling effects on the brief measures used to assess these constructs. Future research should evaluate the utility of alternative measures of acceptability, feasibility, and appropriateness that may not be as susceptible to ceiling effects. More granular quantitative data or qualitative data regarding acceptability and appropriateness can inform future large-scale evaluations of the PEACE implementation toolkit.

These pilot findings provide initial support for the PEACE implementation toolkit as a method for improving EI providers’ caregiver coaching fidelity within community-based early intervention for families of young autistic children. Future directions for research that extend this line of inquiry are needed, including research that expands the sample size, extends the intervention duration, considers the use of more sensitive assessment tools, and explores the effectiveness of the intervention in different contexts.

## Figures and Tables

**Table 1:** PEACE implementation strategies linked to barriers and conceptual frameworks.

Identified Coaching Barrier	Related CFIR Construct	ERIC Strategy	Targeted Construct	PEACE Toolkit Implementation Strategies
Poor provider cohesion and communication; isolation	Inner Setting	Build a Coalition	Norms	Virtual communication platform to enhance communication and social networks among providers
Lack of training	Inner Setting	Develop and Distribute Educational Materials	Self-Efficacy	Self-paced online training videos, tip sheets, and infographics housed in an online resource library
Poor self-efficacy	Individual Characteristics	Facilitation; Consultation; Audit and Feedback	Norms and Self-Efficacy	Group and individual facilitation meetings with auditing of performance and feedback
Poor intentions to implement coaching	Individual Characteristics	Obtain Formal Commitments	Attitudes	Formally setting intentions to coach caregivers with each new client
Beliefs that coaching is a poor fit for some families	Individual Characteristics	Develop Educational Materials	Attitudes	Modules with concrete strategies to support families from minoritized or marginalized backgrounds

**Table 2:** Demographics of participating providers and caregivers.

Variables	Providers N (%)	Caregivers N (%)
Gender		
Female	8 (100%)	8 (100%)
Race		
White	5 (63%)	3 (37%)
African-American or Black	3 (37%)	2 (25%)
Asian	0.0%	1 (12%)
Other	0.0%	2 (25%)
Ethnicity: Latino/Hispanic/Spanish	0.0%	3 (37%)
State		
PA	7 (88%)	7 (88%)
Other	1 (12%)	1 (12%)
Job Title		
Special Instructor	6 (76%)	---
ABA Therapist	1 (12%)	---
Other	1 (12%)	---
Employee Type		
Full-Time Employee	3 (37%)	---
Part-Time Employee	0.0%	---
Independent Contractor	5 (63%)	---
Highest Level of Education		
High School or GED	0.0%	2 (25%)
Some College	0.0%	2 (25%)
College	0.0%	4 (50%)
Graduate/Professional Degree	8 (100%)	0 (0%)
Annual Household Income		
Under USD 20,000	---	4 (50%)
USD 20,000–USD 40,000	---	1 (12%)
USD 40,000–USD 60,000	---	1 (12%)
Over USD 60,000	---	2 (25%)

**Table 3:** Overall coaching fidelity scores at first baseline and final intervention sessions.

Provider	Group	1st Baseline Coaching Fidelity Score	Final Coaching Fidelity Score
1	3	2.82	2.91
2	1	1.09	2.0
3	2	2.43	3.96
4	3	2.23	2.58
5	1	3.0	3.27
6	1	2.76	3.64
7	2	2.55	3.48
8	3	2.77	3.24

**Table 4:** PICCOLO domain scores for caregivers at baseline and after providers completed PEACE group facilitation sessions.

PICCOLO Domain	Minimum	Maximum	Mean	SD
Affection Time 1	9	12	12	1.87
Affection Time 2	10	13	11.8	1.16
Responsiveness Time 1	9	14	12	2.35
Responsiveness Time 2	10	14	12.8	1.64
Encouragement Time 1	8	13	9.6	2.07
Encouragement Time 2	10	13	11.4	1.14
Teaching Time 1	6	9	7	1.41
Teaching Time 2	6	10	7.75	1.70

Note. Affection, Responsiveness, and Encouragement are scored on a 14-point scale, and Teaching is scored on a 16-point scale. Higher values indicate higher rates of supportive parenting behaviors.

## Data Availability

The data supporting the conclusions of this article will be made available by the authors upon reasonable request.
